# Influences of Fiber Volume Content on the Mechanical Properties of 2D Plain Carbon-Fiber Woven Composite Materials

**DOI:** 10.3390/polym16010108

**Published:** 2023-12-29

**Authors:** Jingliang Gong, Nouman Saeed, Xigui Huang, Weiwei Tian, Lixiao Li, Jian Song

**Affiliations:** 1 College of Civil and Transportation Engineering, Shenzhen University, Shenzhen 518060, China; gongjingliang2021@email.szu.edu.cn (J.G.); noumansaeed7846@gmail.com (N.S.); weiweitian410@gmail.com (W.T.); dfsongjian2006@126.com (J.S.); 2 School of Civil and Environmental Engineering, Harbin Institute of Technology (Shenzhen), Shenzhen 518055, China; huang.xigui@foxmail.com

**Keywords:** 2D plain-pattern carbon-fiber woven composites, mechanical performance, fiber volume fraction, progressive damage evolution

## Abstract

The influence of fiber volume content on the mechanical properties of two-dimensional (2D) plain carbon-fiber woven composites is a crucial concern that necessitates immediate attention for large-scale applications in wind turbine blades. In this study, various mechanical tests were conducted on 2D plain carbon-fiber woven composites with different fiber volume contents, and the influences of fiber volume content on the mechanical properties and failure mode of the composite material were analyzed. Using carbon fiber as reinforcement and epoxy resin as a matrix, three types of plates with fiber volume contents of 47%, 50% and 53% were fabricated by using autoclave technology. The tensile, compression and interlaminar shear tests of the two-dimensional woven composites were carried out using MTS series testing machines. The influences of fiber volume content on tensile strength and modulus, compressive strength and modulus, interlaminar shear strength and shear strain energy were investigated. Additionally, the progressive damage development of these two-dimensional woven composites under different stress states was studied using scanning electron microscopy (SEM). The results indicate that the tensile strength and compressive strength increase almost linearly with the increase in fiber volume content, while the interlaminar shear strength increases slowly at low fiber volume content and rapidly at high fiber volume content. The tensile modulus of elasticity slightly increases as the fiber volume content increases, whereas the compressive modulus remains stable at low fiber volume content but gradually decreases at high fiber volume content. With the increase in fiber volume content, the shear strain energy of the specimen increases significantly.

## 1. Introduction

Fiber-reinforced composite material, a blend of fibers and a matrix, provides numerous advantages such as high strength, stiffness, and excellent design performance. It has been extensively utilized in a wide range of fields such as aerospace, civil engineering, energy, and others [1,2,3]. In the energy field, fiber-reinforced composite materials are typically employed in the fabrication of wind turbine blades. The matrix component predominantly comprises epoxy resin, with glass fiber serving as the most common reinforcement [4]. However, the development of large-scale wind turbines has heightened the need for materials that possess greater strength and reduced weight [5,6,7,8]. As a result, the focus has now shifted towards high-strength and lightweight materials for the next generation of composite materials intended for blade fabrication. 

Carbon fiber, exhibiting superior strength and lower density compared to glass fiber, holds the potential to produce lighter and more rigid blades and is anticipated to replace glass fiber in the future [9,10,11]. A comparative study on the tensile properties of carbon-fiber and glass-fiber laminates by Ji [12] revealed an approximately 38.6% higher tensile strength in carbon-fiber laminates. Balokas et al. [13] employed finite element analysis to compare the stress conditions of glass-fiber and carbon-fiber composite materials in wind turbine blades. Their findings indicated a 73.9% reduction in the displacement of carbon-fiber composite materials compared to those made of glass fiber, with the failure stress in all directions also significantly higher than that of glass-fiber composite materials. Mou et al. [14] and Teng et al. [15] both contend that employing carbon fiber in designing the main beam of 122 m long blades can reduce blade weight by 15% to 20%, ultimately leading to a reduction in wind-turbine overall cost by at least 10%.

In addition to the selection of fiber material, the fiber volume content also significantly influences the mechanical strength of composite materials. Pothan et al. [16] investigated the impact of fiber volume fraction on the mechanical properties of glass fabric-reinforced polyester composite materials under static and dynamic loads. Their study revealed that the tensile strength of the material peaked when the fiber volume fraction was 50% and decreased when the fiber volume fraction increased to 70%. Xu et al. [17] employed tensile fatigue tests to compare the woven composite materials of diagonal carbon-fiber epoxy resin with 12,000 and 3000 fiber bundles. Their findings indicated that the fabric with a volume content of 12,000 fibers exhibited higher fatigue strength and static strength than that with 3000 fibers. Christoph et al. [18] examined the effect of fiber volume content on the tensile strength of short carbon fiber-reinforced polypropylene composites. Their results showed that the tensile strength of the material increased with the rise in fiber volume content. Adeniyi and others [19] investigated the effects of different fiber volume contents on the mechanical properties of polystyrene and plant fiber blends and concluded that blends with 20% fiber volume content showed an increase in impact strength of about 27.9% over unfilled blends.

In the production process, the mechanical properties of woven composite materials can vary significantly based on the weaving and forming technologies employed [20]. Two-dimensional (2D) woven materials, which possess strong integrity and excellent shear and impact resistance, are suitable for use as skin materials for wind turbine blades. The fabric’s structural form includes plain, twill, and satin weaves. ‘Plain’ refers to the warp and weft yarns woven alternately with each other [21]. Compared to the other two structural forms, plain weave has the highest frequency of staggered fiber bundles, resulting in better integrity and anti-delamination, as well as greater compactness, than twill and satin weaves. However, the intricate interweaving of warp and weft yarns engenders a complex internal damage mechanism within the material, necessitating the examination of its mechanical properties. While mechanical tests on 2D carbon-fiber woven composites have been conducted by several researchers [22,23,24], comprehensive studies on the mechanical properties of plain-weave carbon-fiber woven composites remain relatively scarce. Furthermore, the influence of fiber content on the material is often overlooked in these studies.

In order to better apply the carbon fiber-reinforced resin matrix material to the laying design of large-scale wind turbine blades, this study intends to systematically investigate the mechanical properties of the composite material with a fiber volume contents range of 47% to 53%, referring to the current fiber volume content of glass fiber-reinforced composites used in DTU 10 MW blades [25]. The layout of this article is ordered as follows: Section 2 explains the preparation and testing methods of the specimens. Section 3 analyzes the experimental results. Subsequently, the main findings of this study are presented in the conclusion in Section 4.

## 2. Materials and Methods

### 2.1. Materials

The experimental material for the prefabricated component incorporates T300-3K carbon fiber (AVIC Composite Cooperation Ltd., Beijing, China) as the reinforcement and bismaleimide QY8911-IV epoxy resin (AVIC Composite Cooperation Ltd., Beijing, China) as the matrix. The fiber content and the number of weaving layers for the two-dimensional plain woven composite materials are detailed in Table 1. In accordance with these weaving parameters, the weaving was entrusted to the Institute of composite materials of Tiangong university (Tianjin, China). The materials were subsequently manufactured by the Beijing Aeronautical Manufacturing Technology Research Institute (Beijing, China) through an autoclave process. Finally, a horizontal milling cutter was employed to cut the prepared two-dimensional plain woven composite material board, thereby obtaining specimens for various experiments.

Prior to testing, the test samples were uniformly numbered in the format A-B-C. Here, ‘A’ represents the type of test, with ‘LS’ indicating a tensile test, ‘YS’ a compression test, and ‘JQ’ a shear test. ‘B’ represents specimens with different fiber volume contents, where ‘1’ signifies 47% fiber volume content, ‘2’ indicates 50% fiber volume content, and ‘3’ denotes 53% fiber volume content. ‘C’ represents the serial number of the specimen under this test, where ‘1’ represents the first specimen, ‘2’ the second specimen, and ‘3’ the third specimen. For instance, ‘YS-3-1’ represents the first compressed specimen with a fiber volume content of 53%. Three specimens were selected for each type of fiber volume content in each experiment to account for potential variability in the experimental results. Additionally, as the two-dimensional plain woven composite material has the same fiber content in its two main directions, the test will only assess the mechanical properties in one direction.

### 2.2. Testing Methods

#### 2.2.1. Tensile Test

**Figure 1:**
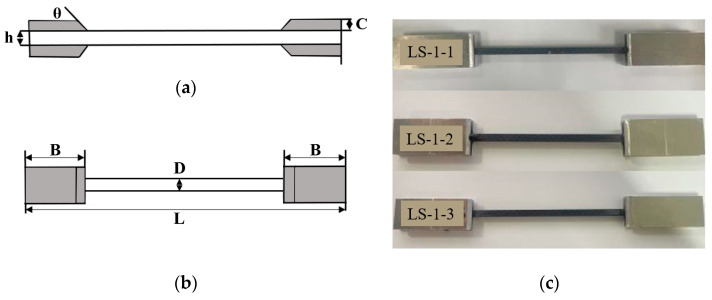
Tensile test specimen (**a**) Front view (**b**) Vertical view (**c**) Partial actual samples.

Figure 1a,b depicts the front and top views of the geometric shape of the tensile test specimens, respectively. The specimens consist of two-dimensional plain woven composite materials with bonded aluminum reinforcement sheets utilizing acrylic transparent AB adhesive. The reinforcement plates are pasted at the ends of composite materials to prevent uneven stress on both ends of the specimen during testing, as well as to protect the clamping end of the specimen from damage prior to testing. In the figure, *L* denotes the length of the specimen, *D* represents the width of the specimen, *B* signifies the length of the reinforcing plate, *h* indicates the thickness of the specimen, *C* refers to the thickness of the reinforcing plate, and *θ* is the oblique angle of the reinforcing plate. Table 2 presents the size parameters of each specimen with different fiber volume contents. Additionally, Figure 1c provides a visual representation of some actual test samples.

**Figure 2:**
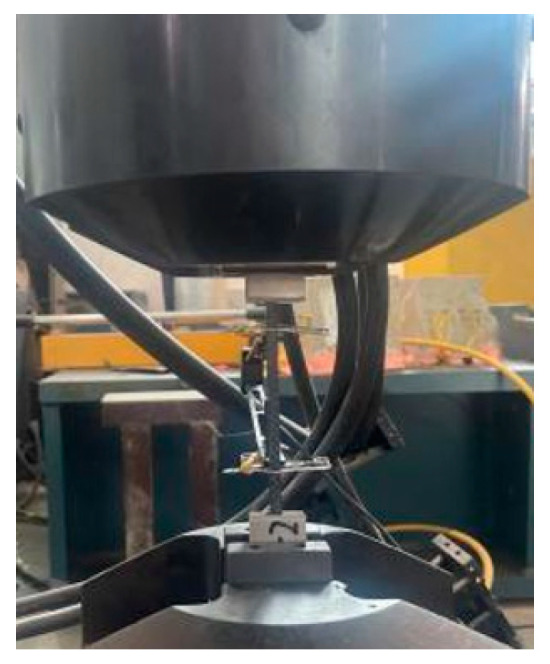
Tensile test diagram.

The tensile stress and tensile strength of each specimen are tested in accordance with the GB/T1447-2005 specification [26]. The experimental apparatus employed was a 50 kN MTS tensile testing machine (MTS Systems Corporation, Eden Prairie, MN, USA), which operated at a tensile speed of 2 mm/min. A static signal testing and analysis system was connected to the testing machine to measure the specimen strain. As shown in Figure 2, during the experiment, we ensured that the fixture heads at both ends clamped 2/3 of the reinforcing plate, and the rest exposed the fixture to avoid shear force on the specimen caused by the reinforcing plate during the clamping process of the fixture.

The tensile strength of the specimen *σ_t_* was calculated by the following equation:

(1) $$\sigma_{t}=\frac{F_{t}}{D\cdot h}$$

where *F_t_* is the maximum tensile stress of the specimen, *D* is the width of the specimen, and *h* is the thickness of the specimen.

#### 2.2.2. Compression Test

**Figure 3:**
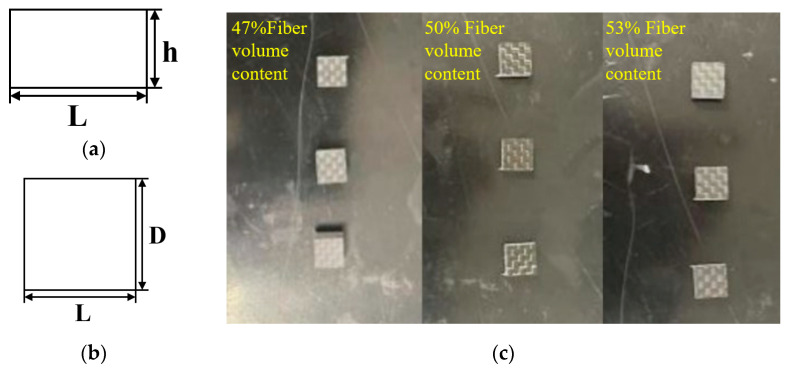
Compression test specimen (**a**) Front view (**b**) Top view (**c**) Actual sample.

The geometric shapes of the two-dimensional plain woven composite material specimen for compression testing are illustrated in Figure 3a,b from the front and top views, with dimensions of 10 ± 0.5 mm for length and width. Figure 3c shows the actual specimen, while the size parameters of each fiber volume content specimen are listed in Table 3.

**Figure 4:**
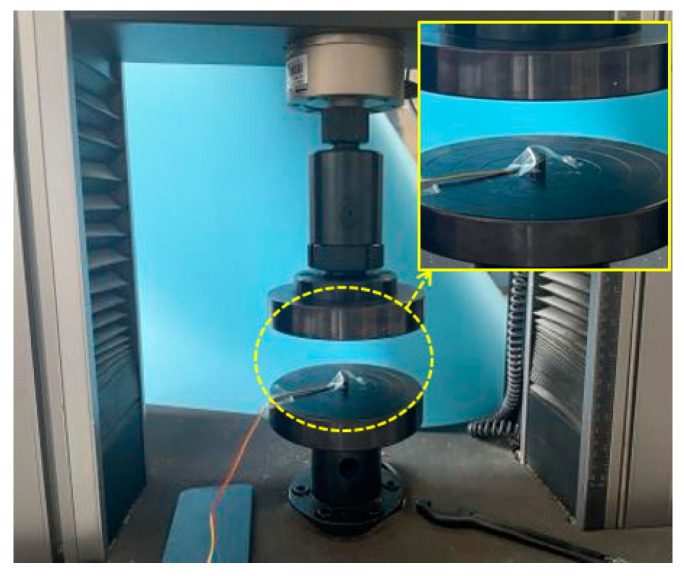
Compression test diagram.

The compressive stress and compressive strength of each specimen are determined in accordance with the GB/T5258-2008 specification [27]. The testing equipment is a 50 kN MTS compression testing machine (MTS Systems Corporation, USA) with a loading speed of 1 mm/min. Since the displacement data collected by the compression machine relate to the downward movement distance of the compression plate, they may not accurately reflect the deformation displacement of the specimen. Therefore, the side of the specimen is polished with sandpaper, and a 3AA strain gauge is glued with 502 adhesive. This gauge is connected to the static signal testing and analysis system, forming a 1/4 bridge three-wire system for the collection of strain data from the specimen, as depicted in Figure 4.

Compressive strength *σ_c_* of the specimen is calculated using the following formula:

(2) $$\sigma_{c}=\frac{F_{c}}{D\cdot h}$$

where *F_c_* is the maximum compressive force of the specimen.

#### 2.2.3. Shear Test

**Figure 5:**
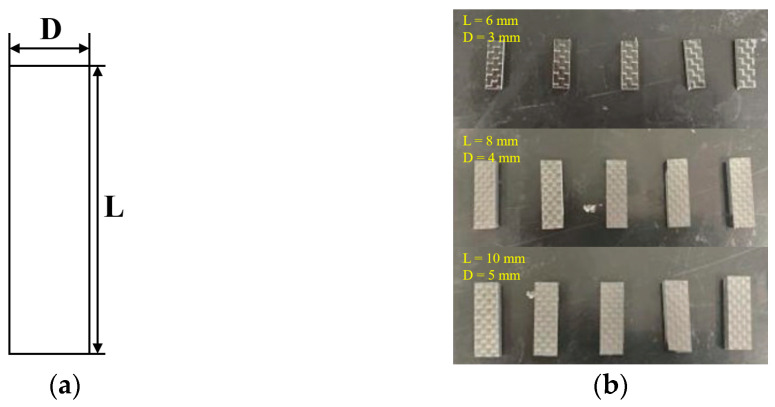
Short-beam shear test specimen (**a**) Top view (**b**) Actual sample.

The shear performance of fiber-reinforced polymer matrix composites is relatively low in comparison to other properties, often determining whether the material experiences premature failure [28]. In this study, a short-beam shear test with relatively accurate measurement results is selected to determine the interlayer shear strength of the specimen. Numerous studies have shown that the magnitude of the span thickness ratio loaded on the specimen directly influences the ultimate failure mode of the specimen, thereby affecting the measurement of interlaminar shear strength [29,30,31]. A larger span thickness ratio results in a smaller interlayer strength value, while a smaller span thickness ratio yields a greater interlayer strength value. Furthermore, once the span thickness ratio falls below a certain limit, classical beam theory is no longer applicable. To ensure accurate results from the short-beam shear method, this study utilizes a shear specimen with a span thickness ratio of 4:1 to prevent bending failure. Figure 5a presents the geometric top view of the interlayer shear specimen. The length, width, and thickness dimensions vary among specimens with different fiber volume contents. Table 4 provides the specific geometric parameters, and Figure 5b displays the actual specimen.

**Figure 6:**
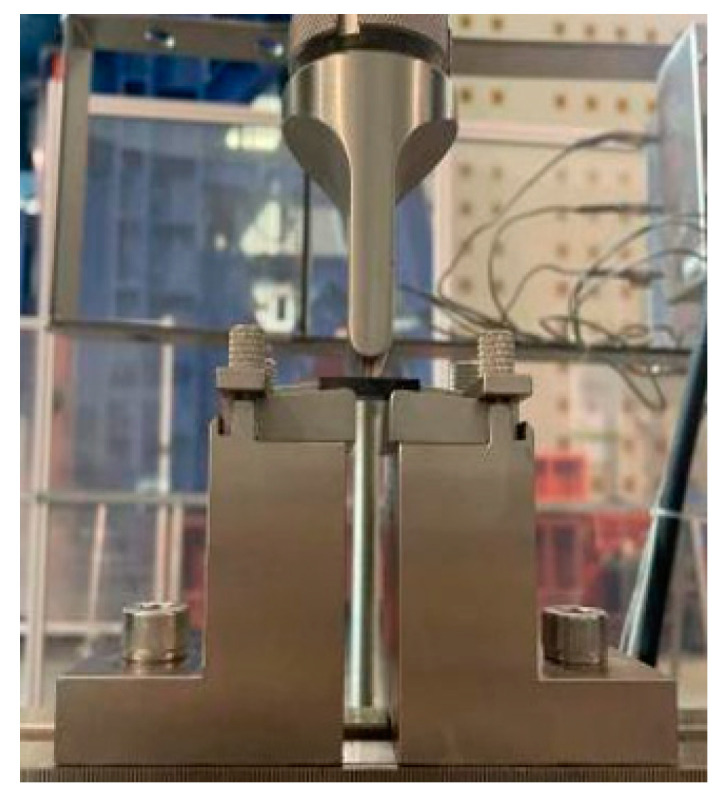
Short-beam shear test diagram.

The shear stress and shear strength of each specimen shall be tested in accordance with specification ASTM D2344-16 [32]. The testing equipment is a 50 kN MTS shear testing machine (MTS Systems Corporation, USA) with a loading speed of 2 mm/min. The clamping of the specimen is depicted in Figure 6.

The shear strength *S* of the specimen is calculated using the following formula:

(3) $$S=\frac{3F_{s}}{4D\cdot h}$$

where *F_s_* is the maximum load of the specimen.

### 2.3. Scanning Electron Microscope (SEM)

**Figure 7:**
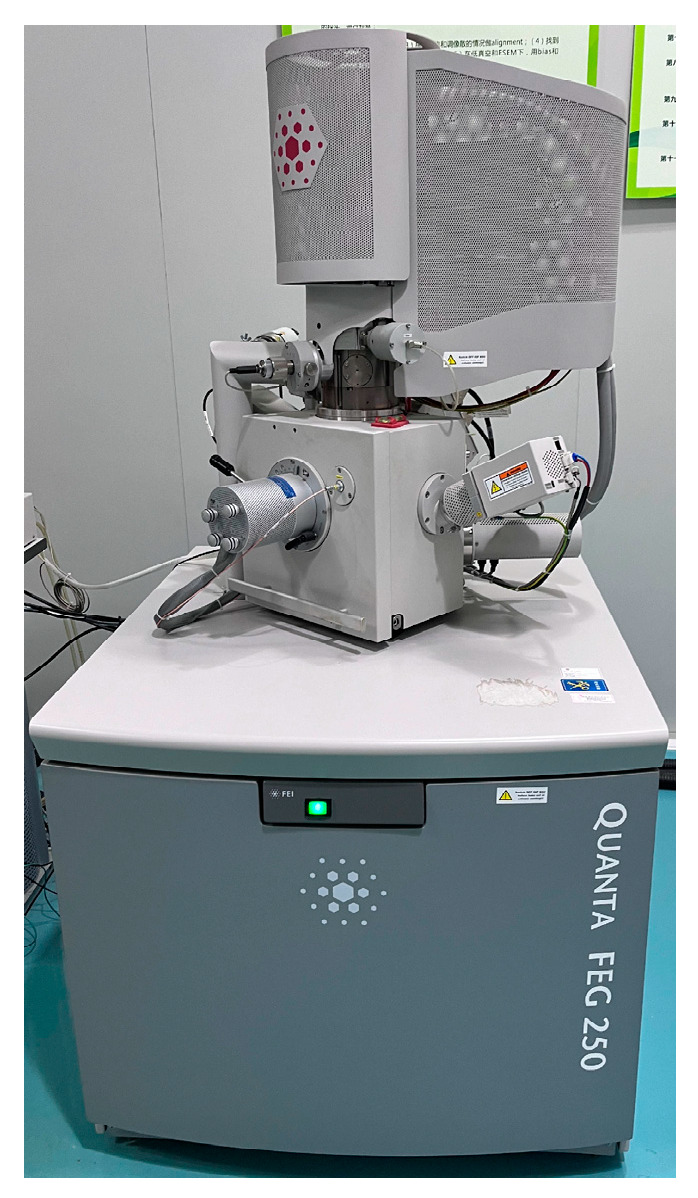
Quanta FEG 250.

A Quanta FEG 250 scanning electron microscope (Field Electron and Ion Company, FEI, Hillsboro, OR, USA) shown in Figure 7) is employed to conduct the micro-analysis of the damage pattern of the specimen. The equipment parameters are shown in Table 5. The samples were cut into small parts using a high-precision cutting machine, left for 24 h to dry, and then sprayed with a conductive film to improve its conductivity. Finally, the processed samples were placed in the sample chamber under vacuum, and we adjusted the focal length and magnification to capture typical images of the damaged specimens.

## 3. Results and Discussion

### 3.1. Tensile Test

**Figure 8:**
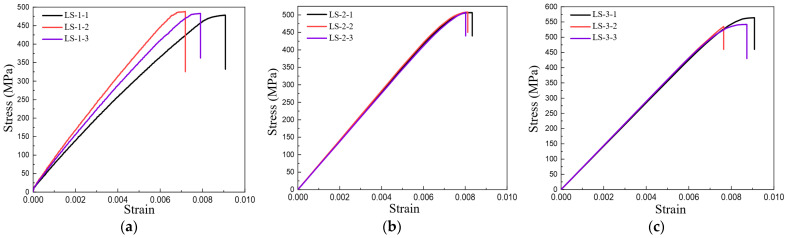
Stress–strain curves of specimens with different fiber volume contents during tensile testing (**a**) 47% (**b**) 50% (**c**) 53%.

Figure 8a–c shows the tensile stress–strain relationship of two-dimensional plain woven composite materials with different fiber volume contents. Overall, the tensile stress–strain behavior of 2D woven composites exhibits a non-linear relationship without a clear yield point. At low stress levels, the tensile stress–strain presents a linear change, but as the material approaches its breaking point, a pronounced non-linear behavior emerges, with strain increasing significantly with stress. This is primarily attributed to a large number of fiber pull-outs and brittle fractures under high-stress conditions. The stress–strain relationships of the three samples with a fiber volume content of 47% are noticeably different; the ultimate stress is similar, but the ultimate strain varies. The stress–strain changes of the three samples with a fiber volume content of 50% are consistent. The LS-3-2 sample, with a fiber volume content of 53%, exhibited brittle fractures during the experiment, which was distinct from the other two samples.

**Figure 9:**
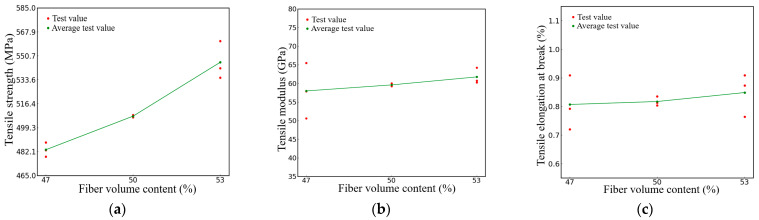
Specimens with different fiber volume contents (**a**) Results of tensile strength (**b**) Results of tensile modulus (**c**) Results of tensile elongation at break.

Figure 9 illustrates the impact of different fiber volume contents on the tensile properties of the specimen. In the figure, the red dot represents the experimental value, and the green dot is the average value of the experimental value under the same volume content. The data depicted in Figure 9a illustrate the relationship between the volume of fiber content and the ultimate tensile strength of the composite material. The average ultimate tensile strength of specimens with a fiber volume content of 50% is 507 MPa, which is 5% higher than that of specimens with a fiber volume content of 47%, while specimens with a fiber volume content of 53% have a tensile strength of 546 Mpa, 7.6% higher than that of specimens with a fiber volume content of 50%. These results suggest a positive correlation between fiber volume content and the tensile strength of materials, as illustrated in Figure 9a. However, the increase in the tensile modulus of the material due to the fiber volume content is relatively small. Figure 9b shows that the tensile modulus of the specimen with 50% fiber volume content only increased by 2.8% compared to the tensile strength of the specimen with 47% fiber volume content. Similarly, the tensile modulus of the specimen with 53% fiber volume content only increased by 3.6% compared to the tensile strength of the specimen with 50% fiber volume content. Figure 9b shows that the average tensile modulus of the specimens with a fiber volume content of 50% is 59.6 Gpa, which is an increase of 2.8% compared to the 47% fiber volume content. The tensile modulus of the specimens with a fiber volume content of 53% is 61.7 Gpa. Figure 9c shows a comparison of the elongation of specimens with different fiber volume contents. The average breaking elongation of the specimens with a fiber volume content of 50% is 0.82%, which is 1.01% higher than that of the specimens with a fiber volume content of 47%. The average breaking elongation of the 53% fiber volume content specimen is 0.85%, which is 3.6% higher than the 50% fiber volume content specimens.

**Figure 10:**
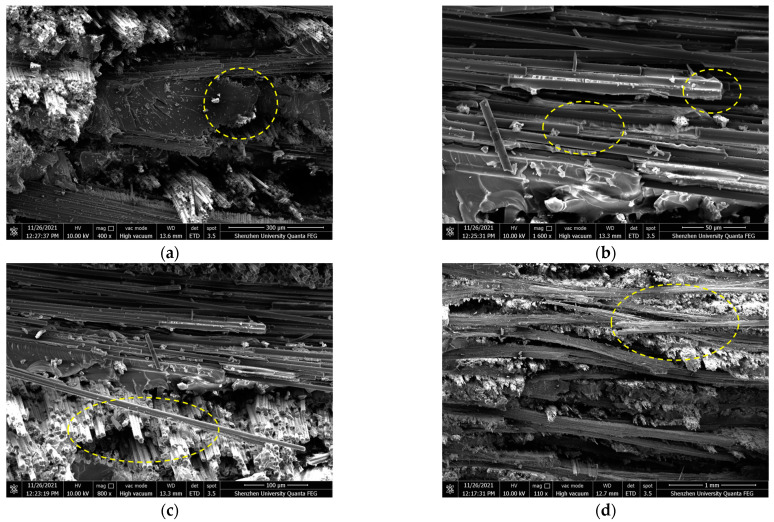
SEM image of the fracture surface of the tensile test piece (**a**) Resin fracture (**b**) Transverse fiber-bundle fracture debonding (**c**) Longitudinal fiber-bundle fracture and extraction (**d**) Fiber delamination fracture.

Figure 10 shows the fracture morphology of the tensile specimen observed under SEM. With the load increases, the cracks in the resin gradually extend until the resin cracks, resulting in the debonding of the interface between the resin and the fiber bundle, transverse and longitudinal fiber-bundle fracture, longitudinal fiber-bundle pull-out, and transverse fiber-bundle delamination fracture. From Figure 10a,c,d, one can observe that the cross-section frequently appears at the intersection of transverse and longitudinal fiber weaving. According to Griffith’s theory for brittle fracture, when there are defects in a material, the stress around the defect will concentrate on the defect under external force and lead to the fracture of the material [33]. Since the composite material is very tight inside, it is difficult for the resin matrix to penetrate the junction, which results in many microcracks. When subjected to tension stress, the concentration of stress leads to the propagation of cracks and eventually induces the material to break.

In fact, during tensile experiments, the specimen experiences sudden fracture once the load reaches a specific point. This implies that the fibers in the specimen are not gradually pulled apart as the load is applied, but rather are pulled apart simultaneously. Upon examining the fracture pattern in Figure 10, it can also be observed that the fracture pattern exhibited by the woven composite material is a flat fracture rather than an oblique fracture. The stress–strain curve of the tensile specimen also indicates that the material’s tensile failure belongs to brittle failure, as the stress suddenly goes straight down once it reaches its peak.

### 3.2. Compression Test

**Figure 11:**
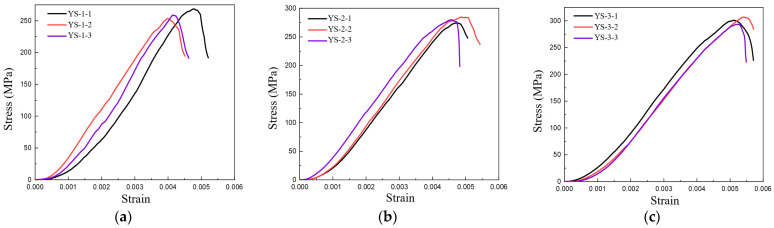
Stress–strain curves of specimens with different fiber volume contents during compression testing (**a**) 47% (**b**) 50% (**c**) 53%.

The stress–strain relationship under compression load for specimens with three different fiber volume contents is shown in Figure 11. From the figure, it can be seen that the stress–strain curve exhibits a slow ‘climbing’ phenomenon in the initial stage, which is due to the lack of bolts at the bottom of the pressure plate of the testing instrument and the presence of an alignment stage on the bottom platform. In the initial stage of loading, the bottom platform continually corrects its alignment position to maintain positive pressure on the compression testing instrument. The compression test instrument is under consistent pressure to ensure accurate measurements. This results in a load-displacement curve that appears relatively flat with minimal elasticity. Furthermore, the stress–strain curve experiences slight fluctuations before reaching its peak. This is due to the fracture strain of carbon fibers being higher than that of the matrix, leading to initial damage to the matrix and a subsequent reduction in the overall load-bearing capacity.

**Figure 12:**
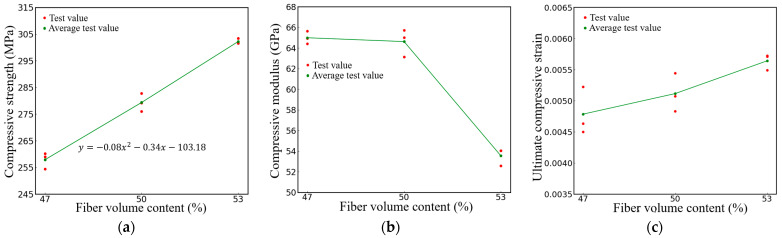
Specimens with different fiber volume contents (**a**) Results of compressive strength (**b**) Results of compressive modulus (**c**) Results of compressive elongation at break.

Figure 12 presents the compression performance of specimens with varying fiber volume contents. As shown in Figure 12a, the mean value of the ultimate compressive strength of the specimen with 50% fiber volume content is 279 MPa, which is 8.32% higher than that of the specimens with 47% fiber volume content, and the average compressive strength of the specimen with 53% fiber volume content is 302 Mpa, 8.2% higher than that of the specimens with 50% fiber volume content. However, the compressive modulus of the composites is negatively correlated with the fiber volume content, as shown in Figure 12b. The average compressive modulus of the 50% fiber volume content specimen is 64.6 Gpa, which is slightly lower than that of the 47% fiber volume content specimens. From the fitting results and experimental values, it can be noted that with the increase in fiber volume content, the elastic modulus may reach the maximum and then gradually decrease. When the fiber volume content increases from 50% to 53%, the average compressive modulus of the material decreases significantly from 64.6 Gpa to 53.6 Gpa. The increase in the fiber volume content also has a certain impact on the ultimate compressive strain of the composites, as shown in Figure 12c. The average ultimate compressive strain of the 50% fiber volume content specimens, which is 0.0051, increased by 6.25% compared to the 47% fiber volume content specimens. The average compressive limit strain of the 53% fiber volume content specimens, which is 0.00564, increased by 10.3% compared to the 50% fiber volume content specimens.

**Figure 13:**
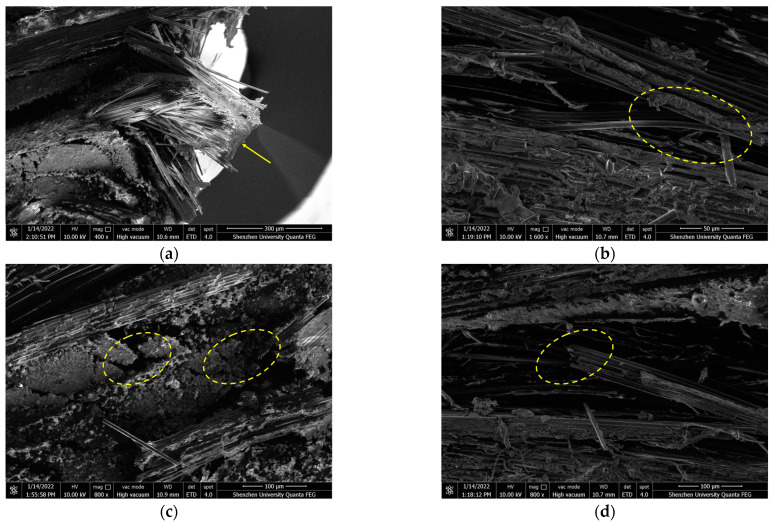
SEM image of fracture surface of compression test piece (**a**) Longitudinal fiber-bundle debonding at the loading surface (**b**) Longitudinal fiber-bundle delamination (**c**) Resin fracture (**d**) Transverse fiber-bundle delamination.

Figure 13 shows the SEM image of the compressed specimen after failure. As observed in Figure 13a, the debonding of the interface between the longitudinal fiber bundle and the resin matrix primarily happens at the loading surface’s end, which leads to delamination. The delamination phenomenon propagates inward along the longitudinal direction towards the material’s interior as the loading force continuously increases. As depicted in Figure 13b, the longitudinal fiber bundle remains intact and undamaged under the compressive load, while a substantial delamination between the fiber bundles exposes them. With further increase in the load, the resin matrix and transverse fiber bundles start to fracture, as exemplified in Figure 13c,d.

### 3.3. Short-Beam Shear Test

**Figure 14:**
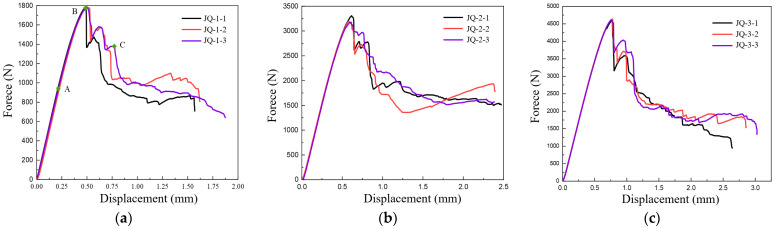
Load-displacement curve of specimens with different fiber volume contents in shear test (**a**) 47% (**b**) 50% (**c**) 53%.

Figure 14a–c illustrates the relationship between shear load and displacement for specimens with three distinct fiber volume contents of 47%, 50%, and 53%, respectively. The figures reveal that prior to the shear load reaching its peak value, the variation of shear load and shear displacement for the three specimens, each with the same fiber volume content, exhibits good consistency. The shear load and shear displacement essentially demonstrate an approximate linear relationship. However, when the shear load reaches its peak value, indicating the onset of interlayer shear failure in the fiber, the relationship between load and displacement among different specimens exhibits certain disparities. The interlaminar shear failure of specimens with varying fiber volume contents demonstrated good ductility and effective energy dissipation capacity.

**Figure 15:**
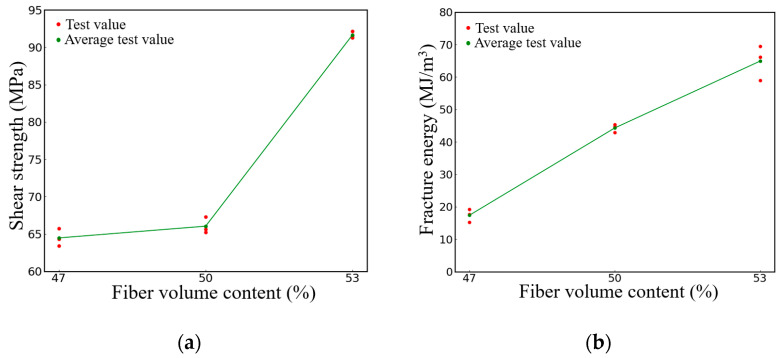
Specimens with different fiber volume contents (**a**) Results of shear strength (**b**) Results of fracture energy.

Figure 15 presents the test results of the shear performance of the composite materials with three different fiber volume contents. As depicted in Figure 15a, the shear strength of the specimen with a 50% fiber volume content increased by only 2.4% compared to the specimen with a 47% fiber content. However, when the fiber volume content reaches 53%, the shear strength increased by 42.2% compared to the specimen with a 47% fiber content, marking a significant increase compared to that of the specimen with a 50% fiber content. Figure 15b shows the relationship between the shear fracture strain energy and fiber volume content of the two-dimensional woven composite. The energy absorbed by composite materials per unit volume, from the undamaged state to the point of fracture, can be determined by calculating the area enclosed by the load-displacement curve and the horizontal axis, which represents the fracture energy [34]. According to Figure 15b, the fracture energy of the specimens with a 53% fiber volume content is 64.9 MJ/m^3^, which is 46.6% higher than the 44.3 MJ/m^3^ fracture energy of the specimens with 50% fiber volume content, and 273% higher than the 17.4 MJ/m^3^ fracture energy of the specimens with 47% fiber volume content. Obviously, the fracture energy of the specimen increases gradually as fiber volume content increases.

**Figure 16:**
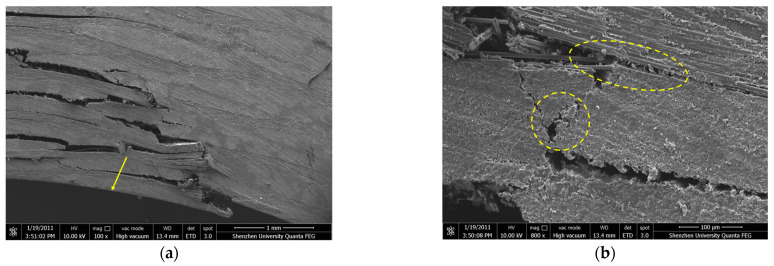

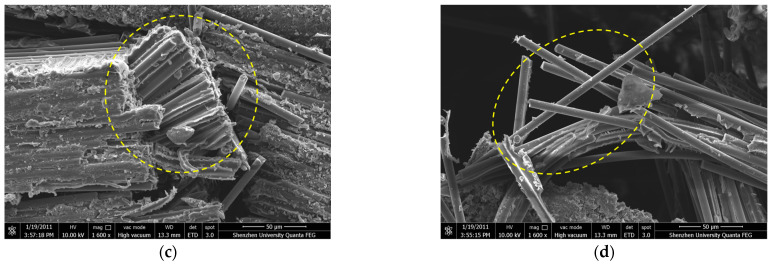
SEM image of the fracture surface of the shear test piece (**a**) Resin cracking on the loading surface (**b**) Longitudinal fiber-bundle delamination and resin cracking (**c**) Fracture and debonding of warp and weft fiber bundles at the overlap (**d**) Tensile surface fiber-bundle fracture.

Figure 16 presents the SEM image of the shear failure specimen. When combined with the load-displacement curve of the shear test in Figure 14a, it can be observed that when the specimen reaches damage initiation point A near the elastic stage, the upper loading surface incurs severe damage, as depicted in Figure 16a. The crack extends from the contact surface of the loading head in an octagonal shape to both sides, with the resin exhibiting cracking and fiber-bundle detachment, as shown in Figure 16b. At this juncture, there are no cracks on the cross-section along the width direction of the specimen, which is due to a sudden drop in load caused by the short-term occurrence of cracks. Consequently, it can be inferred that the crack on the upper loading surface is the root cause of the specimen’s damage, which also results in the localization of shear strain near the test loading place. As cracks resulting from transverse fiber-bundle cracking propagate through the thickness of the material to intersect with interwoven fiber bundle, they cause damage and debonding at the overlap of warp and weft yarns. This leads to the process of progressive damage to the test sample.

When the bearing capacity further rises to near the top point (B) of the load-displacement curve, the failure mode of the test piece is mainly the breaking and debonding of the longitudinal and latitudinal fiber-bundle overlap, as shown in Figure 16c, and the tensile fracture of the fiber bundle at the bottom, as shown in Figure 16d. The specimen continues to experience failure at increasing bearing capacities. Subsequently, it can be observed that the overall load shows a downward trend, but in some stages, as shown by point C in the figure, the bearing capacity briefly increases. This is because after some fiber bundles inside the specimen fail, other intact fiber bundles can still redistribute the force and continue to bear the load.

## 4. Conclusions

Two-dimensional woven composites, characterized by their light weight, high strength, and good flexibility, make them a promising material for offshore wind turbine blade structures. The mechanical properties of woven composite materials can vary significantly depending on the weaving and forming technologies employed. This study systematically investigates the influences of fiber volume contents on the mechanical properties of two-dimensional plain-woven composites. Tensile tests, compression tests, and short-beam shear tests were conducted on composites with fiber volume contents of 47%, 50%, and 53%. The evaluated mechanical properties include tensile strength, compressive strength, laminar shear strength, and corresponding elastic moduli. The progressive damage process of the 2D woven composites was analyzed in detail based on the SEM test. The main results are as follows:

(1) The tensile strength of 2D woven composites increases approximately linearly with the increase in fiber volume content. The average tensile strengths of specimens with fiber volume contents of 47%, 50% and 53% were 483 MPa, 507 MPa and 546 MPa, respectively. The change of longitudinal tensile elastic modulus with fiber volume content is relatively small. The tensile elastic moduli corresponding to 47%, 50% and 53% fiber volume contents were 58 GPa, 59.6 GPa and 61.7 GPa, respectively. The ultimate tensile strain of two-dimensional woven composites was relatively discrete, with the average value ranging from 0.82% to 0.85%.
(2) The ultimate compressive strength of the two-dimensional woven composites increased almost linearly with the increase in fiber volume content. The average ultimate compressive strength and average compressive modulus of the 2D woven composites with 47% fiber volume content were 258 MPa and 65 GPa, respectively. The average ultimate compressive strength of 2D woven composites with 50% fiber volume content was 279 MPa, and the corresponding compressive modulus was 64.6 GPa. The average ultimate compressive strength of the two-dimensional woven composite with 53% fiber volume content was 302 MPa, and the corresponding average compressive modulus was 53.6 GPa.
(3) The shear strength of the 2D woven composites increased slightly at lower fiber volume contents and increased significantly at higher fiber volume contents. The average value of the shear strength of specimens with 47%, 50% and 53% fiber volume contents were 64.5 MPa, 66.1 MPa and 91.7 MPa, respectively. The shear strain energy of the specimens with 53% fiber volume content was 64.9 MJ/m^3^, which is 46.6% higher than that of the 50% fiber volume content specimen at 44.3 MJ/m^3^, and 273% higher than that of the 47% fiber volume content specimen at 17.4 MJ/m^3^.

This research provides valuable insights into the mechanical behavior of two-dimensional woven composites, which can guide the design and application of these materials in various fields, particularly in offshore wind turbine blades.

## Figures and Tables

**Table 1:** The weaving parameters and plate dimensions of two-dimensional plain woven composite materials.

Number of Fiber Layers	Fiber Monolayer Thickness (mm)	Fiber Volume Content (%)	Material Size (Length × Width × Thickness/mm)
15	0.125	47%	200 × 126 × 4
20	0.125	50%	200 × 126 × 5
25	0.125	53%	200 × 126 × 6

**Table 2:** Dimensions of specimens with different fiber volume contents during tensile testing.

Fiber Volume Content (%)	*L* (mm)	*D* (mm)	*h* (mm)	*C* (mm)	*B* (mm)	*θ* (°)
47	200	5 ± 0.5	4	2.5	50	45
50	200	5 ± 0.5	5	2.5	50	45
53	200	5 ± 0.5	6	2.5	50	45

**Table 3:** Size of specimens with different fiber volume contents during compression test.

Fiber Volume Content (%)	L (mm)	D (mm)	h (mm)
47	10 ± 0.5	10 ± 0.5	4
50	10 ± 0.5	10 ± 0.5	5
53	10 ± 0.5	10 ± 0.5	6

**Table 4:** Size of specimens with different fiber volume contents in short-beam shear test.

Fiber Volume Content (%)	L (mm)	D (mm)
47	6 ± 0.5	3
50	8 ± 0.5	4
53	10 ± 0.5	5

**Table 5:** Parameters of SEM equipment.

Resolution Ratio	Accelerating Voltage	Magnification
1.0 nm@30 kV, 3.0 nm@1 kV	200 V~30 kV	14–1,000,000×

## Data Availability

Data are contained within the article.
